# Pediatric Nicotine Exposures Reported to US Poison Centers

**DOI:** 10.1001/jamanetworkopen.2026.0479

**Published:** 2026-03-04

**Authors:** Perry E. Rosen, Danielle R. Bartsche, James B. Leonard, Howard A. Greller, Bruce E. Ruck, Diane P. Calello

**Affiliations:** 1 New Jersey Poison Information and Education System, Newark; 2 New York Institute of Technology College of Osteopathic Medicine, Old Westbury, New York; 3 Rutgers New Jersey Medical School, Department of Emergency Medicine, Newark; 4 Washington Poison Center, Seattle

## Abstract

This cross-sectional study investigates trends in pediatric nicotine exposures by product type and exposure route from 2016 to 2023 in the US.

## Introduction

Recent analyses have described trends in pediatric nicotine ingestions, offering insight into exposures involving newer products, such as nicotine pouches.^1^ Although poison center surveillance continues to report pediatric nicotine exposures annually,^2^ no national study has assessed trends across all routes of exposure since 2016,^3^ when cartridge- and disposable-based e-cigarettes became prominent.^4^ Unintentional nicotine exposures occur predominantly in children younger than 5 years, particularly exploratory toddlers aged 1 to 2 years.^1,3^ We analyzed national poison center data from 2016 through 2023 to characterize trends in pediatric nicotine exposures across product types and exposure routes.

## Methods

This retrospective cross-sectional study used the National Poison Data System (NPDS) to identify single-substance nicotine exposures in children aged 1 month to 5 years reported from January 1, 2016, through December 31, 2023. All reasons for exposure were included except those coded as confirmed nonexposures or unrelated effects; all fatalities were retained regardless of relative contribution to fatality designation. Products were categorized as traditional tobacco, e-cigarettes and liquid nicotine, or other nontobacco nicotine (eTable in Supplement 1). NPDS definitions and case-coding procedures are standardized nationally.^2^ Descriptive statistics and odds ratios (ORs) for health care facility use were calculated; trends were assessed with linear regression, with significance set at a 2-sided *P* < .05. This study followed the STROBE reporting guideline and was deemed exempt from review and consent by the Rutgers University institutional review board under 45 CFR §46.104(d)(4) as secondary research involving analysis of existing, deidentified data that was deemed minimal risk.

## Results

Among 92 973 reported pediatric nicotine exposures (91.2% among children aged ≤2 years; median [IQR] age, 1.25 [0.92-2.00] years; 45.3% female, 54.5% male, and 0.2% unknown sex), 99.6% were unintentional. Traditional tobacco product exposures decreased from 9122 in 2016 to 5204 in 2023 (−43.0%; *P* < .001), whereas e-cigarette exposures increased from 2044 to 7009 (242.9%; *P* < .001). Ingestion was the predominant route of exposure (80 054 exposures); however, inhalation-based exposures increased from 70 in 2016 to 5292 in 2023 (7460.0%; *P* < .001) (Figure). Inhalation exposures were primarily associated with e-cigarettes and liquid nicotine products (Table).

**Figure.**
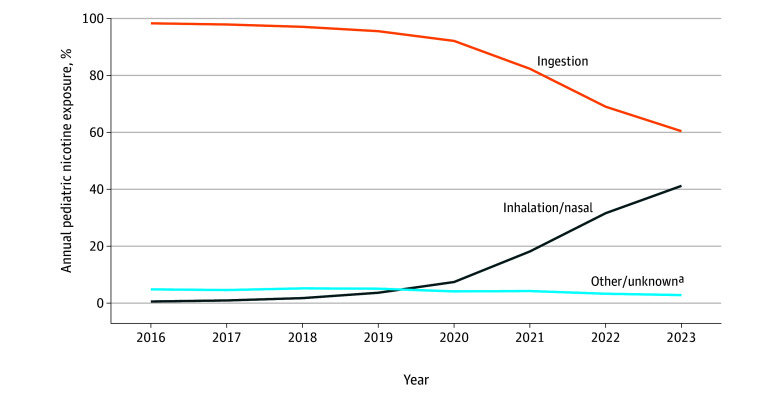
Annual Distribution of Pediatric Nicotine Exposures by Route, 2016-2023 Routes of exposure were not mutually exclusive; a single exposure (among 92 973 total exposures) could involve more than 1 route (96 717 total routes). Percentages were calculated using the total number of exposures per year as the denominator. ª Other/unknown exposure routes include dermal, ocular, otic, parenteral, rectal, vaginal, and unspecified routes.

**Table.** Product Type Among Pediatric Nicotine Exposures by Route, 2016-2023

Route of exposure	Exposures, No. (%) (N = 92 973)^a^			Total, No.
	Traditional tobacco	E-cigarettes and liquid nicotine	Other nontobacco nicotine	
Ingestion	56 797 (70.9)	16 732 (20.9)	6525 (8.2)	80 054
Inhalation or nasal	1467 (11.5)	11 102 (87.4)	142 (1.1)	12 711
Other or unknown^b^	1806 (45.7)	1921 (48.6)	226 (5.7)	3953

^a^ Percentages represent the proportion of route-specific mentions attributable to each product type. Percentages may exceed 100% within rows because routes of exposure are not mutually exclusive. A single exposure could involve more than one route.

^b^ Other or unknown exposure routes include dermal, ocular, otic, parenteral, rectal, vaginal, and unspecified routes.

While 98.9% of exposures resulted in no or minor effects or were lost to follow-up, 1023 resulted in moderate effect, 31 in major effect, and 2 in deaths (both e-cigarette liquid ingestion). Health care facility evaluation occurred in 16.8% of cases, more often with e-cigarettes and nicotine liquid (OR, 1.24; 95% CI, 1.19-1.29) and other nontobacco nicotine (OR, 1.27; 95% CI, 1.18-1.36) compared with traditional tobacco.

## Discussion

Nicotine exposure in children younger than 6 years changed measurably over this cross-sectional study’s period. E-cigarette exposures increased 242.9%, whereas traditional tobacco exposures decreased by 43.0%. Prior research suggested that the Child Nicotine Poisoning Prevention Act of 2015 was associated with a reduction in e-cigarette–related exposures reported to US poison centers, citing a decline from January 2015 to April 2017.^5^ Our findings indicate that this trend reversed, coinciding with the market shift toward disposable and cartridge-based e-cigarettes introduced after 2015.^4^ Notably, the increase in e-cigarette exposures persisted despite federal actions raising the minimum purchase age in 2019 and restricting flavored cartridge-based e-cigarette sales in 2020.

Recent prior studies are limited to ingestion and therefore underestimate the true pediatric burden.^1^ Inhalation exposures increased substantially, suggesting a transition from traditional ingestion hazards to direct device-related exposures in which children activate devices themselves. Developmentally typical exploratory behaviors and the brightly colored appearance of many e-cigarette products may facilitate these exposures.^6^ However, this study is also limited by reliance on poison center data, which are subject to underreporting and other systematic biases.

This distinction underscores the need for surveillance that captures evolving product formats and exposure routes. Device-level safety regulations analogous to those required for liquid-nicotine packaging under the Child Nicotine Poisoning Prevention Act of 2015, including flow restrictors or designs that limit pediatric device activation, may help reduce these preventable events.

## Notes

Supplement 1. eTable. Composite Categorization of Generic Nicotine-Based Products

Supplement 2.
Data Sharing Statement
