# Error in Results

**DOI:** 10.1001/jamanetworkopen.2026.18594

**Published:** 2026-05-22

In the Research Letter titled “Pediatric Nicotine Exposures Reported to US Poison Centers,”^1^ published March 4, 2026, the statement in the Results section that “98.9% of exposures resulted in no or minor effect” should have stated that 98.9% of the exposures had no or minor effect or were lost to follow-up. The article has been corrected.^1^
