# Supplementary material for: Pediatric Nicotine Exposures Reported to US Poison Centers
Source: JAMA Netw Open. 2026 Mar 4;9(3):e260479. doi: 10.1001/jamanetworkopen.2026.0479 (PMC12961511; doi:10.1001/jamanetworkopen.2026.0479)
Supplement: Supplement 2. — Data Sharing Statement [file jamanetwopen-e260479-s002.pdf]

## Data Sharing Statement

Rosen. Pediatric Nicotine Exposures Reported to US Poison Centers. J*AMA Netw Open*. Published online March 4, 2026. doi:10.1001/jamanetworkopen.2026.0479

### Data

**Data available:** No

### Additional Information

**Explanation for why data not available:** Data requests must be forwarded to the National Poison Data System.
